# Hungry Ghosts Eat Casino Chips: Associations Between Dispositional Greed and Gambling

**DOI:** 10.1177/01461672251315200

**Published:** 2025-03-12

**Authors:** Joshua Weller, Marcel Zeelenberg, Barbara Summers

**Affiliations:** 1 University of Leeds, UK; 2 Tilburg University, The Netherlands; 3 Vrije Universiteit Amsterdam, The Netherlands

**Keywords:** risk-taking, gambling, decision-making, impulsiveness, dispositional greed

## Abstract

Dispositional greed is characterized as the insatiable desire for more. Although greed may be a driving force for wealth accumulation, it can also relate to increased financial difficulties and risk-taking. Across two studies in different countries, The Netherlands (Study 1, *N* = 1,118) and England, Study 2, *N* = 4,855), we tested the degree to which dispositional greed was associated with gambling outcomes. Greedy individuals reported greater gambling participation and more negative gambling-related consequences. Moreover, Study 2 found that greed was associated with maladaptive gambling-related cognitions (e.g., cognitive distortions, positive expectations, and the perceived inability to stop) beyond that explained by trait motor impulsiveness. In addition, dispositionally greedy individuals reported being more focused on financial motivations for gambling, having greater confidence in winning, and less concern with realized losses. The current study demonstrates links between dispositional greed and risk-taking in a real-world context, highlighting biased decision-making cognitions for greedy individuals.

“*Gambling operates under the premise that greed can be satisfied by luck*.”

-Rita Mae Brown

*“Let’s make lots of money.”*

-Pet Shop Boys

Gambling, for most partaking in it, is a pleasurable activity that provides entertainment and enjoyment. In recent years, gambling opportunities have proliferated, largely associated with the rapid growth of the online gaming industry. Nowadays, one can gamble via online gaming apps, including virtual casinos, sportsbooks, and daily fantasy sports. In the United States alone, gambling revenue took in $54.9 billion in 2022, breaking the record set in 2021 by 13.5% (American Gaming Association, 2023). Similarly, countries, such as the United Kingdom and the Netherlands have also posted large gross gambling yields £15.1b (in the year to March 2023) and €1.0b revenue from online gambling (projected for 2024), respectively (Gambling Commission, 2023; Statistica, 2024). In contrast to those who gamble responsibly, some individuals may struggle with gambling and realize associated adverse psycho-social consequences. In the United Kingdom, for example, over 2 million adults are thought to experience some level of gambling harm (Gambling Commission, 2022). Problem gambling is associated with increased debts, dissolution of interpersonal relationships, and losses may exacerbate mood disorders and other harmful behaviors like alcohol, substance abuse, and suicide attempts (Wardle et al., 2018). At the pathological level, the *Diagnostic and Statistical Manual of Mental Disorders* (5th ed.; *DSM-5*; American Psychiatric Association, 2013) defines gambling disorder as persistent and recurrent problematic gambling behavior leading to clinically significant impairment or distress, as indicated by the individual exhibiting four (or more) of the following in a 12-month period, including but not limited to: gambling preoccupation, repeated attempts to control one’s gambling, needing to gamble with more money to achieve the desired excitement, chasing losses (i.e., gambling more to break-even), and borrowing money to pay back gambling debts.

It follows that better understanding of not only who gambles excessively, but also what psychological processes may perpetuate these behaviors, is vital for promoting responsible gambling as well as developing potential interventions to reduce problem behavior. Numerous studies examined associations between personality and problem-gambling behavior, and these have largely focused on higher-order trait dimensions, such as the Big Five (Dudfield et al., 2023; Strømme et al., 2021), or on narrower traits related to risk-taking, such as those related to sensation-seeking and self-control (e.g., Canale et al., 2015; Cyders & Smith, 2008; Michalczuk et al., 2011). In contrast, the current study examines the idea that dispositional greed (Zeelenberg & Breugelmans, 2022) is associated with gambling behavior. Dispositional greed is a relevant, but overlooked, trait with respect to gambling, not only for its notable associations with the desire to acquire excessive resources (Seuntjens, Zeelenberg, Breugelmans, & Van de Ven, 2015; Seuntjens, Zeelenberg, Van de Ven, & Breugelmans, 2015), but also because of its links with impulsiveness (Seuntjens et al., 2019; Seuntjens, Zeelenberg, Van de Ven, & Breugelmans, 2015) and risk-taking (Li et al., 2019; Mussel et al., 2015; Mussel & Hewig, 2016; Rodrigues et al., 2023). However, research has neither extensively examined the associations between greed and real-life risk-taking, nor the psychological mechanisms that may account for such effects.

We investigated these issues in nationally representative community samples in two European nations (i.e., the Netherlands and England). Across both studies, we predicted that higher levels of dispositional greed would be positively associated with various indicators of gambling behavior and realized harmful consequences. In Study 2, we additionally tested the degree to which dispositional greed was associated with maladaptive gambling-related cognitions (GRCs; Raylu & Oei, 2004), such as positive expectancies of the gambling experience and control beliefs, accounting for trait differences in impulsiveness.

## Dispositional Greed

Greed has a long history of being associated with socially undesirable behaviors (Zeelenberg et al., 2025). For instance, Christianity refers to greed as one of the “deadly sins.” Similarly, Buddhism refers to “hungry ghosts,” reborn beings who led a previous life characterized, in part, by greed; these beings are said to have “large stomachs and tiny mouths,” ever unable to quench their desires (Rotman, 2021). Conversely, some have lauded the consequences of greed, as it is thought to result in economic growth, which might generate a surplus that benefits society (Bruhn & Lowrey, 2012; Oka & Kuijt, 2014).

Dispositional greed is defined as dissatisfaction with one’s current state, combined with the insatiable desire for more of any valued entity (Seuntjens, Zeelenberg, Breugelmans, & Van de Ven, 2015). People reporting low greediness tend to be satisfied with what they have, and not seek more. In contrast, those scoring high on greediness experience dissatisfaction with current possessions and are the most likely to display a variety of acquisitive behaviors. Although money and financial gains are associated with dispositional greed, other resources, such as power, status, sex, and food could also be a target of greed (Hoyer et al., 2025; Weiß et al., 2024). For instance, greed was related to hoarding behavior during the COVID-19 pandemic in Japan (Yoshino et al., 2021). In addition, being greedy is associated with wanting more friends (Seuntjens, Zeelenberg, Van de Ven, & Breugelmans, 2015), and with a higher number of sex partners (Hoyer et al., 2024).

Several scales measure differences in dispositional greed, demonstrating strong convergent validity and similar external validity (Mussel et al., 2018; Zeelenberg et al., 2022). The Dispositional Greed Scale (DGS; Seuntjens, Zeelenberg, Van de Ven, & Breugelmans, 2015; Zeelenberg & Weller, 2025) is the most widely used scale, and validated for application in numerous languages and cultures. Converging evidence across various scales and samples suggests that levels of dispositional greed are normally distributed in the population (Krekels & Pandelaere, 2015; Zeelenberg & Breugelmans, 2022).

Dispositional greed is related to, but separable from, traits that reflect some form of “wanting more,” such as materialism, envy, and self-interest (Crusius et al., 2021; Krekels & Pandelaere, 2015; Seuntjens, Zeelenberg, Breugelmans, & Van de Ven, 2015; Seuntjens, Zeelenberg, Van de Ven, & Breugelmans, 2015). For instance, materialism is often conceptualized as a value (Richins, 2004), while greed represents a desire to obtain more, regardless of whether the target is a material entity. Greed differs from envy in that it stems mostly from wanting more (internally motivated), whereas envy is mostly driven by wanting what others have (externally motivated; Seuntjens, Zeelenberg, Breugelmans, & Van de Ven, 2015). In addition, Hoyer et al. (2025) found that greed and self-interest share many of the negative relationships with important life outcomes, but greed was positively related to household income, while self-interest was negatively related. Greed was also positively related to the number of sexual partners, whereas self-interest was unrelated.

Dispositional greed has also been related to broad personality dimensions (e.g., the Big Five), in which the general finding is that greedier people are less agreeable and more neurotic (Krekels & Pandelaere, 2015; Mussel & Hewig, 2016; Sekhar et al., 2020; Seuntjens, Zeelenberg, Van de Ven, & Breugelmans, 2015). Extraversion, openness, or conscientiousness did not show a consistent relationship with greed. The HEXACO honesty–humility dimension includes a facet labeled greed avoidance, which reflects the desire to acquire material goods and status; thus, this scale reflects perceived happiness with materialistic goods, but not aspects of acquisition or dissatisfaction (Ashton & Lee, 2007). Other studies find that greedier people are more impulsive and have less self-control (Seuntjens et al., 2019; Seuntjens, Zeelenberg, Van de Ven, & Breugelmans, 2015), are more easily tempted (Hoyer et al., 2023), and score higher on psychopathic and other dark triad traits, (Mussel & Hewig, 2016; Sekhar et al., 2020; Veselka et al., 2014).

## Greed, Risk-taking, and Gambling

The degree to which dispositional greed is associated with real-life risk behaviors, and specifically gambling, remains an open question. However, converging indirect evidence supports this assertion. First, some studies found an association between greed and risk-taking using controlled experimental tasks. Mussel et al. (2015) reported that greedy individuals showed lower neural responses to losses (vs. gains), compared with less greedy counterparts. Similarly, Hoyer et al. (2023) reported that greed was more strongly associated with greater expected benefits from engaging in risky behaviors across different domains, rather than lower risk perceptions. In addition, that study reported that dispositional greed was significantly associated with maladaptive risks, rather than recreational or social risks. Second, dishonest and unethical behaviors associated with greed (Zeelenberg et al., 2025) may promote symptoms of problem gambling, namely, concealing gambling losses or gambling frequency. For instance, research has found that positive greed attitudes and motivations were associated with lying and cheating in pursuit of self-interest (Piff et al., 2012).

Third, related traits also hint toward associations between dispositional greed and gambling. Weller and Thulin (2012) reported that lower HEXACO-Greed Avoidance (and the broader honesty/humility dimension; Weller & Tikir, 2011) was associated with greater risk-taking (with hypothetical gambles) for both potential gains and potential losses (although, see Seuntjens, Zeelenberg, Van de Ven, & Breugelmans, 2015, for null effects in a hypothetical mixed gamble paradigm). Furthermore, A. B Carver and McCarty (2013) found that materialistic values were endorsed by the heaviest casino gamblers, compared with other gambling sub-types. In addition, Eyzop et al. (2019), comparing 65 pathological gamblers with 65 matched non-problem gamblers, reported that individuals endorsing materialistic values gambled more for financial motives and were more likely to excessively gamble (cf., Estévez et al., 2021). Similarly, HEXACO honesty/humility predicts problem-gambling behavior severity (Kim et al., 2019; Lee et al., 2013; McGrath et al., 2018; Mishra et al., 2018). Finally, dispositional greed is associated with financial motivations (Seuntjens et al., 2016, 2019), which often are also reasons for gambling (Floyd et al., 2024; Tabri et al., 2022).

## The Current Research

The picture that emerges is one of greed as an impulsive, opportunistic, anti-social, and egoistic trait, rather than a beneficial one. Integrating these insights with the insatiability and acquisitiveness that are central to the greed construct, we propose that greedy people may be drawn to gambling, because it offers the potential to satisfy their acquisition goals in the quickest possible time. Subsequently, greedy individuals who *do* gamble, may do so more often and may report greater negative consequences associated with it. Across large community samples from the Netherlands (Study 1) and England (Study 2), we tested the associations between dispositional greed and gambling behaviors, such as poly-gambling activities and problem severity.

## Study 1

### Method

#### Participants

Participants in this study were participants of the Longitudinal Internet Studies for the Social Sciences (LISS panel; www.lisspanel.nl), a true probability sample of the Dutch population which is administered and managed by the non-profit research institute Centerdata (Tilburg University, The Netherlands). Data collection for this sample occurs monthly, with subsets of the entire panel (over 7,000 participants) being invited to complete studies and experiments. Participants in this panel receive a monetary incentive for each completed questionnaire. In the current study, we used two separate datasets from LISS: (a) data involving dispositional greed, collected in 2013 (Seuntjens et al., 2013) and (b) data involving gambling behaviors, collected in 2017 (Meerkerk, 2017). Only participants who completed both studies were included in the present study (*N* = 1,118, 51.8% female). The majority of participants had a Dutch background (60.4%), 24.5% reported being a first- or second-generation Dutch resident of Western background, 15.1% reported being a first- or second-generation Dutch resident of non-Western background. The mean age of the sample was 55.24 years (*SD* = 15.96), 93.1% reported achieving at least the equivalent of U.S. high school diploma and reported a mean monthly gross income of €2,700.

#### Measures^1^

##### Dispositional Greed Scale

We used the seven-item DGS (Seuntjens, Zeelenberg, Van de Ven, & Breugelmans, 2015). Sample items include, “As soon as I have acquired something, I start to think about the next thing I want,” “I always want more” (1 = strongly disagree, 5 = strongly agree); *M* = 2.01, *SD* = 0.73, Cronbach’s α = .88.

##### Materialism

We used the Material Values Scale (Richins & Dawson, 1992). Only the nine highest loaded items on the original scale were collected (see Seuntjens, Zeelenberg, Van de Ven, & Breugelmans, 2015). Sample items include “Buying stuff gives me a lot of pleasure” (1 = strongly disagree, 5 = strongly agree); *M* = 2.47, *SD* = 0.66, Cronbach’s α = .80.

##### Gambling Participation

Participants were asked: “In the past 12 months, have you participated in ANY form of gambling? This includes, but not limited to, lottery tickets, scratch cards, bingo, horse racing, sport betting, and casino gambling?” For those who responded “Yes” (63.8%), several follow-up questions were asked to characterize their gambling behavior, namely:

###### Gambling Types

Participants were provided with a list of nine common gambling activities (i.e., lottery draws, scratch cards, bingo, slot machines in pubs/restaurants/casinos, online slots/instant wins, horse/dog race betting, sports betting, in-person or virtual casino games, poker) and were asked to whether they engaged in this behavior over the past 12 months. We created a poly-gambling engagement variable by summing the number of endorsed activities (range = 0–9).

###### Problem-Gambling Severity

Negative consequences related to gambling were measured by the South Oaks Gambling Screen (SOGS; Lesieur & Blume, 1987), resulting in a score from 0 to 20. Scores 3 to 4 = some gambling risk, 5 or greater suggests presence of pathological gambling.

### Results and Discussion

In this sample, 63.8% of respondents gambled on any game of chance within the past 12 months (see Table 1). Lotteries were the most popular gambling type (57.7%) and 63.5% of the respondents who gambled reported that lotteries were their only form of gambling. Scratch cards were the second most popular gambling activity (14.5%), while the other activities had endorsement rates of 5.7% or less. The overall sample played, *M =* 0.93 (*SD=* 0.98) different types of games (range = 0–7), *M* = 1.46, *SD* = 0.86, for those who reported gambling within the past 12 months. SOGS risk scores ranged from 0 to 3, *M* = 0.37, *SD* = 0.50. No participants met the established threshold for probable pathological gambling risk and only three (0.3%) scored > 1. Resultantly, we do not consider this measure any further.

**Table 1.** Gambling Participation by Type and Frequency Within the Last 12 Months.

Gambling type	Study 1 (*N* = 1,118 Dutch citizens)	Study 2 (*N* = 3,297 U.K. citizens)	
	% participated	% participated	% participated monthly or more
National lottery draws	57.7	86.7	58.3
Scratch cards	14.5	68.4	34.5
Fruit/slot machines (in pubs/restaurants/casinos)	4.8	38.8	14.1
Online slot machine games	–	40.2	23.1
Bingo	5.7	44.0	18.5
Horse/dog races	0.0	43.3	17.5
Sports betting	2.0	51.8	31.3
Casino games (virtual or in person)	4.3	31.1	14.8
Poker at a pub/club	2.1	20.9	9.6

Dispositional greed and materialism were positively correlated, *r* = .64, *p* < .001. We then examined the correlations between dispositional greed, materialism, and poly-gambling behavior (square-root transformed to reduce skewness) for those who gambled within the past 12 months. Dispositional greed (*r =*.16, *p <* .001) and materialism (*r =*.12, *p* = .002) were both associated with poly-gambling behavior. A subsequent linear regression found that dispositional greed uniquely accounted for poly-gambling variance (*B* = 0.16, *p* < .001), holding materialism (*B* = 0.01, *p* = .86) constant, *F* (2, 711) *=* 9.89, *p* < .001.

Finally, we explored whether dispositional greed would be stronger for those who gambled via activities other than solely lottery draws, which are typically lower stakes than other gambling activities, in addition to lotteries. An independent-samples *t*-test comparing lottery-only gamblers with other gamblers revealed significant mean-level differences in both dispositional greed (*M*_lottery only_ = 1.96, *SD* = 0.71; *M*_poly-gamblers_ = 2.20), *SD* = 0.74), *t*(711) = 4.34, *p <* .001. *d* = 0.34 and materialism (*M*_lottery only_ = 2.43, *SD* = 0.64; *M*_poly-gamblers_ = 2.61), *SD* = 0.63), *t*(711) = 3.52, *p <* .001. *d* = 0.27, with the effects being stronger for greed.

These results provide preliminary evidence that dispositional greed is associated with gambling behavior, above and beyond individual differences in materialism. These effects suggest that dispositional greed may be associated with seeking out more gambling opportunities. However, the low base rates of both actual gambling behavior other than lotteries and prevalence of problem-gambling severity in this sample limit our ability to make firm conclusions. In addition, it is important to note that although the endorsement of gambling was assessed, the frequency of each activity was not, further limiting the conclusions.

## Study 2

The purpose of Study 2 was fourfold. First, because greed and gambling measures in Study 1 were 4 years apart, we sought to examine these associations in a larger, contemporaneous sample. Second, the Dutch sample did not include problem gamblers. It is notable that, at the time of the Dutch assessment, online gambling was not yet legal in the Netherlands (only becoming legal in late 2021). To address these limitations, we tested these associations in a larger study, in a larger gambling market, namely, England, which has a much more established gambling culture and industry and is currently the largest gambling market in Europe in terms of online gross gambling revenues (European Gaming & Betting Association, 2022). Third, we tested the degree to which maladaptive GRCs (Raylu & Oei, 2004), would be associated with greed. Finally, because Study 1 revealed that materialism did not account for the association between gambling and greed, we turned our attention to the degree to which greed-gambling associations were independent of individual differences in impulsiveness.

### Maladaptive GRCs

Although many greedy individuals may never gamble, for those that do, we propose that those with high dispositional greed may be more likely to develop maladaptive GRCs that may perpetuate gambling once engaged, and potentially exacerbate problems. The problem-gambling literature suggests that a host of maladaptive cognitions predict problem-gambling severity (e.g., Blaszczynski & Nower, 2002; Goodie & Fortune, 2013; Leonard et al., 2021; Shaw et al., 2023). Some of these beliefs can be considered cognitive distortions, relating to over-reliance on associative processing, such as endorsing the gambler’s fallacy, the “hot-hand” bias, the belief in illusory correlations, in which the context of gambling may be the belief that lucky objects may influence gambling outcomes, or memory biases that may selectively forget losses and highlight wins (Scoboria & Wilson, 2011). In addition, self-serving biases may also operate, such an illusion of control (Langer, 1975), in which an individual’s perceived abilities make them feel in control of random events.

Raylu and Oei’s (2004) Gambling-Related Cognition Scale (GRCS) is the most widespread in the literature and includes five separate maladaptive cognitions. The *illusion of control* scale reflects beliefs that irrelevant factors, such as luck, can influence gambling outcomes, whereas the *predictive control* scale relates to erroneous beliefs about one’s skill level, and also includes rational decision-making errors, such as those present with gambler’s fallacy and hot-hand biases. *Interpretative bias* reflects an individual’s tendency to reappraise gambling outcomes in a manner that would continue gambling, including memory biases for losses, or attributing losses to bad luck. Other cognitions in the GRCS may not directly reflect cognitive errors or biases but may indirectly reflect biased thinking. The *gambling expectancies* scales ascribe accentuated positive motivational beliefs about gambling experiences, in which the gambler feels happier when gambling, or tries to reduce stress through gambling. As Raylu and Oei (2004) note, if an individual views gambling in a positive way and as the only means to cope with stresses of daily life, it may lead to rationalization for continuing gambling, seeing it as a primary outlet for supporting their happiness. Finally, the GRCS includes an *inability to stop* scale, which assesses the strength of one’s confidence in stopping gambling, which is believed to be an important thought distortion when predicting problem-gambling severity and relapse (Raylu & Oei, 2002; Smith et al., 2015). Although this particular belief may not correspond directly to cognitive errors per se, it reflects the acknowledgment that colder cognitive influences (e.g., stepping away from a gambling table when one is ahead, not gambling beyond one’s means, etc.) may not be in balance with more affective, experiential cues like chasing wins or chasing losses to reduce one’s sense of loss aversion.

Research suggests that these cognitions are associated with over-reliance on automatic/experiential processing (Toplak et al., 2007). Emond and Marmurek (2010) found that individuals who reported greater tendencies to engage in experiential (vs. rational) thinking styles demonstrated greater maladaptive GRCs. Similarly, Fletcher et al. (2011) found that lower analytic thinking was related to gambling biases and superstitious thinking (cf., Leonard & Williams, 2018). Conversely, Armstrong et al. (2020) found that rational thinking styles predicted protective gambling cognitions, which subsequently were associated with decreased problem-gambling severity.

Yet, no research has examined whether dispositional greed is associated with biased judgments and decisions. However, several lines of evidence suggest this may be the case. It is reasonable to speculate that the pursuit of acquiring more is accompanied by an expectancy of success. Greedy people may find greater positive expected benefits in activities which have high payouts while discounting the riskiness of a situation (Hoyer et al., 2023). Another possibility is that greedy people embark on accumulation behaviors because they are optimistic about their chances, manifesting either as a general tendency to feel optimistic about the future (C. S. Carver & Scheier, 2009), or as the presence of optimistically biased cognitions, namely, interpretive biases and illusions of control.

In addition, traits that comprise greed’s nomological network have been associated with judgment and decision-making errors. Traits, such as (low) honesty–humility and disinhibition, are associated with lower decision-making competence (Garofalo et al., 2021; Weller et al., 2021). Similarly, dispositional greed has been associated with traits, such as egoism and self-interest, which would promote biased, egocentric thinking (Hoyer et al., 2025; Krekels & Pandelaere, 2015; Seuntjens, Zeelenberg, Breugelmans, & Van de Ven, 2015; Seuntjens, Zeelenberg, Van de Ven, & Breugelmans, 2015). Direct associations have also been observed between impulsiveness and both GRCs and gambling severity (MacLaren et al., 2015; Navas et al., 2017), while low honesty/humility has been associated with greater coping motivations for gambling (McGrath et al., 2018). Using a measure adapted from the GRCS, Ching et al. (2016) found that materialism was related to maladaptive compulsive buying-related cognitions.

### Method

This study was approved by the host university’s Ethical Review Board (#LTLUBS-396). Study methodology was pre-registered and available through Open Science Framework https://osf.io/y85jr/.

#### Participants

We recruited participants through a third-party crowdsourced research firm (Cint) as part of a larger project to examine nationwide gambling behavior in England. In addition to a general population sample, the survey firm identified potential participants with prior stated interest in gambling, thus also obtaining an additional sub-sample. To be eligible for this study, a participant had to be 18 years of age or older and a resident of England. Quotas based on U.K. Census estimates for region, age and gender were established.

#### Data Cleaning

We followed data cleaning procedures specified in the preregistration. We first examined responses for evidence of careless responding (e.g., taking less than 5 minutes to complete the survey, evidence of careless responses sets, self-reporting that they did not carefully or honestly answer the questions). We further used the *r* package *careless* to identify potential outliers, which calculates indices of careless responding (Yentes & Wilhelm, 2018), such as *maximum longstring values* and *intra-individual response variability* indices that are beyond a gap in a distribution, *Mahalanobis distances* that are beyond a “gap” in the distribution can be excluded. In addition, univariate outliers, *z*-scores of >|3| and a disconnection from the rest of the distribution were similarly excluded at the variable level. Cases that were missing more than 15% of responses were excluded listwise.

There were 9,003 survey clicks on the invitation which advertised a study about personality and gambling, with 6,016 of these clicks agreeing to participate after reading the participant information sheet. Decisions to handle outliers were made a priori, based on considerations and remedies suggested by Pickering and Blaszczynski (2021), who highlighted potential challenges for collecting problem-gambling data in convenience samples (see also Chandler & Paolacci, 2017; Lovett et al., 2018). We removed participants’ data who abandoned the survey (*n* = 296), those who showed clear evidence of straight-lined responding throughout the survey (*n* = 147), and those who self-stated that they did not respond honestly or carefully (*n* = 87). Continuing to follow our data retention criteria (e.g., completion duration < 5 minutes, using the *r careless* package to further identify problematic cases, such as low variability/straight-lining), we removed 703 additional participants. We retained a final sample size of *N* = 4,783 (*n*_general population_ = 3,869 and *n*_prior gambling interest_ = 914). Median age was 48 years, 50.1% male, 49.2% female, 0.5% transgender/non-binary/preferred to self-describe, 0.2% did not report. Participants were primarily of White-U.K. origin (87.6%); 3.0% reported Black/African/Caribbean ethnicity, 0.3% Asian, 2.6% mixed ethnicity, 0.5% reported other ethnicity, and 6.1% did not respond. With respect to annual income, 22.3% of participants reported earning £20,000 or less per year, 35.0% between £20,000 and £39,999, 21.0% between £40,000 and £59,999, 15.5% over £60,000, and 6.2% did not report.

We examined the degree to which the two subsamples differed from each other. The gambling interest subsample contained slightly more males, χ^2^(1, 4,750) 3.71, *p* = .05, 53.3% male vs. 46.6% female in the subsample, 50%-50% in the general population sample). We also observed a significant difference in the age between these two groups, *t*(4,775) = 3.02, *p <* .01; however, the mean difference across the two groups was trivial, 48.15 vs. 46.22, for the general and gambling samples, respectively. There were no significant differences for education level, eta = .02, household income (eta = 0.00; region of residence (Cramer’s *V* = .05, *p* = .09), or ethnicity, χ^2^(1, 4,753) = 0.98, *p* = .32, 11.5% and 12.8% non-White participants, respectively).

#### Measures

We included the following measures in this study^2^:

##### Dispositional Greed

We used the shortened, three-item version of the DGS (Seuntjens et al., 2016). “As soon as I have acquired something, I start to think about the next thing I want,” “I always want more,” and “Actually, I am kind of greedy” (1 = strongly disagree; 5 = strongly agree), *M* = 2.57, *SD* = 0.81, Cronbach’s α = .63.

##### Impulsiveness

We included five items from the Abbreviated Barratt Impulsiveness Scale (Coutlee et al., 2014): “I am self-controlled,” “I concentrate easily,” “I act on impulse,” “says things without thinking,” and “I plan trips well ahead of time” (1 = strongly disagree to 5 = strongly agree). Because there has been debate regarding whether scores on this scale should be combined as a sum score, or if subscale (i.e., motor, attention, and non-planning) scores should be used (see Coutlee et al., 2014), we conducted an exploratory factor analysis with oblimin rotation, on a random sample of 50% of the participants. The results suggested a two-factor solution: a two-item motor impulsiveness scale (Imp-Motor; *M =* 2.98, *SD* = 0.97; *r =* .57) and a three-item scale that included attention and non-planning items (Imp-A/NP; *M =* 2.30, *SD* = 0.71; α = .63).^3^

##### Gambling Cognitions and Behavior

###### Gambling Participation

Participants were asked: “In the past 12 months, have you participated in ANY form of gambling? This includes, but not limited to, lottery tickets, scratch cards, bingo, horse racing, sport betting, and casino gambling?” For those who responded “Yes” (67.1%), we asked several follow-up questions to better characterize their gambling behavior, namely:

###### Amount Spent

We asked participants to estimate approximately how much they have spent (in GBP) on gambling activities within the last 14 days. If they had not gambled within this time frame, they were instructed to answer 0. Reported amounts that exceeded three standard deviations above the mean (*n* = 12; *M =* 151.58, *SD* = 4,855.14) were winsorized to £2,000.

###### Gambling Types and Frequency

Participants were asked to report their gambling frequency (0 = never, 1 = less than 6 months, 2 = every other month, 3 = monthly, 4 = weekly, 5 = daily) for nine common gambling activities (i.e., lottery draws, scratch cards, bingo, slot machines in pubs/restaurants/casinos, online slots/instant wins, horse/dog race betting, sports betting, in-person or virtual casino games, poker). A mean gambling frequency was then computed. In addition, we finally created a poly-gambling engagement variable by first coding any frequency response greater than 0 as 1 (Yes), and then adding the number of endorsed activities (range = 0–9), as in Study 1 (range = 0–9).

###### Problem-Gambling Severity Index

Negative consequences related to gambling were measured by the nine-item Problem-Gambling Severity Index (PGSI; Ferris & Wynne, 2001), answered on a 4-point scale (never, sometimes, most of the time, almost always). Scores are summed, ranging from a total score from 0 to 27. Scores > 8 represent problem gambling, 3 to 7 represent moderate level of gambling problems, and 1 to 2 represent gamblers with a low level of problems.

###### Gambling-Related Cognitions

We asked participants who reported gambling within the last 12 months, to complete the GRCS (Raylu & Oei, 2004). The GRCS assesses five domains of GRCs (1 = strongly disagree to 4 = strongly agree): gambling expectancies (four items, *M* = 2.01, *SD* = 0.74, α = .85), illusion of control (four items, *M* = 1.65, *SD* = 0.76, α = .88), predictive control (six items, *M* = 1.89, *SD* = 0.70, α = .86), inability to stop gambling (two items,^4^ *M* = 1.59, *SD* = 0.82), and interpretive bias (four items, *M* = 1.91, *SD* = 0.75, α =.84).

#### Additional Exploratory Measures

We also considered the following measures that were part of the larger project to gain a broader characterization of the association between greed and gambling.

##### Gambling-Related Variables

We asked participants the following (all on: 1 = strongly disagree to 4 = strongly agree): (a) three-item attitudes toward ***winning*** (“My past wins prove that I will be successful at gambling in the long-run,” “I expect to win more than lose when gambling over the long-run” and “I make sure to tell others when I’ve won gambling”); (b) three-item ***loss chasing*** (“If I lose one gamble, it’s best to double the wager the next time,” “If I’ve already lost that day, making more risky bets sounds like the best way to break-even or get ahead,” and “If I would lose a gamble, I would bet more next time to break even”); (c) a six-item scale about ***general attitudes toward losing*** (“Losses are not very stressful or upsetting to me”; see Supplementary Information for full items), *M* = 2.32, *SD* = 0.63, α = .76. Higher scores reflect a greater tendency to shrug off losses, and not experience distress because of them; and (d) two items from the Gambling Motives Questionnaire (Dechant, 2014) related to different ***financial motivations***, “I gamble to win money” and “I gamble because worried about not winning if I don’t play.” Participants completed these items on a 3-point scale (1 = not a reason, 2 = somewhat a reason, 3 = very much a reason).

##### Individual Differences

We asked the following (1 = strongly disagree to 4 = strongly agree): (a) ***Regret***, measured by Schwartz et al.’s (2002) five-item Regret scale (e.g., “When I think about how I’m doing in life, I often assess opportunities I have passed.”), *M* = 3.10, *SD* = 0.74, α = .72 and (b) a one-item ***risk-taking*** measure, “I consider myself to be a risk-taker,” *M* = 2.05, *SD* = 0.87.

#### Data Analytic Plan

We first conducted descriptive statistics and correlational analyses for the variables of interest. Using Mplus 8.6 (Muthén & Muthén, 2017), we then adopted a structural equation modeling (SEM) approach to test the unique contributions of dispositional greed in explaining the variance in (a) PGSI scores, (b) behavioral indicators of gambling, and (c) GRCs, holding impulsiveness, and sociodemographic covariates constant.^5^ Parameters were estimated using a weighted least squares mean and variance adjusted (WLSMV) estimator, a type of diagonally weighted least squares (DWLS) estimator, for analyses involving the PGSI due to the non-normality associated with the scale. For the other analyses, we used a maximum-likelihood with robust estimator (MLR) method. Model fit was evaluated using root mean square error of approximation (RMSEA), standardized root mean square residual (SRMR), Tucker–Lewis index (TLI), and comparative fit index (CFI). Variances for latent variables were fixed at 1. To reduce model complexity, direct paths that did not demonstrate a zero-order correlation > .|10| were not included in the tested model (Gignac & Szodorai, 2016).

Due to being part of a larger data collection, we performed a sensitivity power analysis in PowerSEM v0.1.2 (Wang & Rhemtulla, 2021) for the three-item dispositional greed latent variable predicting a nine-item PGSI latent variable. Factor loadings were conservatively set at .6 for each latent variable. We assumed a two-tailed test (α= .01), with 1,000 simulations. The current sample size would provide > 99.0% power to detect a direct effect of at least .10, with 95% of the parameter estimates falling between .06 and .14.

### Results and Discussion

#### Gambling Behavior

We first examined the gambling tendencies of the current sample (Table 1). Over the past 12 months, 67.1% of participants reported gambling, in any form, at least once. Most participants gambled online vs. in person (61.5% mostly or always online vs. 22.7% mostly/always in-person). Across the nine activities, 50.6% of gamblers reported participating in at least four activities (*M* = 4.25, *SD =*2.76). The most common gambling activities were purchasing a ticket for a national lottery draw and instant-win scratch cards. Within the last 14 days, gamblers reported spending a median of £10, with 7.3% spending over £100 during this period.

The mean PGSI score for those gambling within the last 12 months was 3.5 (*SD* = 5.73). According to suggested cut-scores for risk levels (Ferris & Wynne, 2001), 27.6% scored 3 or higher, suggesting at least a moderate level of gambling-related problems leading to some negative consequences, and 17.6% reported a score of 8 or higher.

#### Correlation Analyses

Table 2 shows the correlations for the variables of interest. Neither age, gender, nor education level were associated with any gambling participation within the last 12 months. However, for those who did gamble during this period, age was inversely associated with poly-gambling activities, mean gambling frequency, amount spent over the past 14 days, and PGSI scores. In contrast, reported household income level was positively associated with all outcomes except PGSI scores. Education level was positively associated with poly-gambling activities and mean gambling frequency. Gender did not correlate with the gambling outcomes above *r = .|*10,*|* with the exception that men were more likely to spend more on gambling within the past 14 days than women.

**Table 2.** Correlations Between Study Variables in Study 2.

Variable	1	2	3	4	5	6	7	8	9	10	11	12	13	14	15
1. Age	—														
2. Gender^a^ (male = 0)	−.22**	—													
3. Education level	−.16**	−.04	—												
4. HH income level	−.16**	−.04	.32**	—											
5. Greed	−.39**	−.01	.12**	.15**	—										
6. Impulsiveness-motor	−.21**	−.01	−.06*	−.01	.36**	—									
7. Impulsiveness-A/NP	−.18**	.03	−.10**	−.09**	.07**	.24**	—								
8. GRC-Inability to Stop	−.32**	.07**	.07**	.03	.37**	.27**	.04	—							
9. GRC Expectancies	−.25**	−.10**	.06**	.04	.33**	.23**	−.03	.62**	—						
10. GRC Illusion of Control	−.38**	.00	.11**	.07	.38**	.27**	.00	.76**	.71**	—					
11. GRC Predictive Control	−.35**	−.02	.08**	.06	.38**	.26**	.01	.68**	.76**	.82**	—				
12. GRC Interpretive Bias	−.35**	−.07**	.11**	.08	.40**	.26**	.02	.68**	.78**	.76**	.83**	—			
13. Poly-gambling (sum)	−.39**	−.01	.13**	.14**	.30**	.24**	.04	.53**	.53**	.56**	.54**	.55**	—		
14. Mean gambling frequency	−.29**	−.10**	.11**	.14**	.31**	.24**	−.04	.58**	.58**	.59**	.57**	.58**	.87**	—	
15. £ spent gambling last 14 days (winsorized)^a^	−.11**	−.18**	.02**	.11**	.18**	.15**	−.01	.40**	.43**	.34**	.36**	.40**	.49**	.64**	—
16. PGSI score^a^	−.41**	−.03	.09**	.05	.35**	.32**	−.16**	.66**	.48**	.56**	.54**	.59**	.55**	.54**	.42**
17. Any gambling last 12 months? (1 = yes)	−.05	−.03	.02	.12**	.12**	.05**	.00	—	—	—	—	—	—	—	—

*Note*. With the exception of the binary gambling participation variable, N = 3,029 to 3,207 for gambling-related correlations; otherwise N = 4,480 to 4,776. Spearman’s rho correlations reported for amount spent and PGSI scores **p < .01. ^a^Spearman’s rank-order correlation.

Consistent with the predictions, dispositional greed was positively associated with gambling participation. Holding demographic variables constant, a one-unit increase in dispositional greed was associated with a 31.6% increase in the odds for gambling within the past 12 months (*B* = 0.28, *p* < .001; 95% confidence interval [CI] for odds ratio 1.20–1.44). Dispositional greed was also positively associated with poly-gambling, gambling frequency, and PGSI scores. Moreover, as in Study 1, gamblers who played other games than solely lottery draws reported greater dispositional greed (*M =* 2.67, *SD* = 0.80) than lottery-only players (*M =* 2.36, *SD* = 0.80), *t*(3,205) = 7.47, *p <* .001, *d =* 0.40. Similarly, greater impulsiveness was positively associated with gambling indicators for those reporting recent gambling, but not for gambling participation. In contrast, optimism was only associated with lower PGSI scores.

Also supportive of the hypotheses, greed was positively associated with impulsivity. However, it was more strongly associated with the motor subscale than the attention/non-planning scale (which had a very small effect size). Greed, and motor impulsiveness, but not the Imp-A/NP scale, were both positively associated with all five GRCSs.

#### Associations Between GRCs and Gambling Outcomes

As expected, all five GRCSs were positively associated with the primary gambling outcomes. Greater endorsement of gambling-related cognitions were associated with higher PGSI scores, poly-gambling behavior, gambling frequency, and amount spent in the past 14 days. The correlations for all GRCS subscales were relatively uniform across the assessed outcomes.

#### SEM Analyses

**Figure 1.**
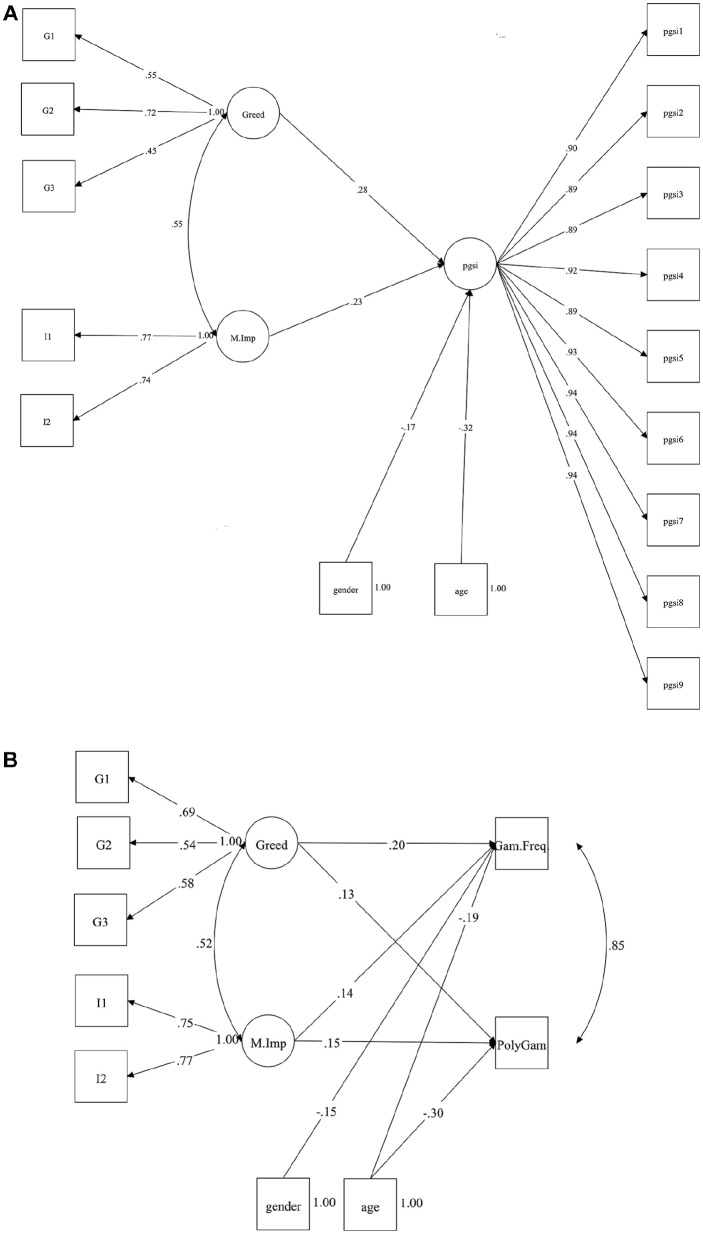
SEM Results by Outcome in Study 2. (A) PGSI. (B) Behavioral Indicators. *Note*. Only variables with significant direct paths to outcome > = .|10| shown in figures. M.Imp = Motor Impulsiveness Gam.Freq = mean gambling frequency; Polygambling = sum of different types of gambling reported.

We next tested the degree to which greed uniquely accounted for variance in the gambling outcomes, holding impulsiveness, and covariates constant (see Supplemental Online information SI-2 for parameter estimates for all variables included). Figure 1A shows the significant path coefficients for the SEM predicting PGSI scores. Model fit statistics showed a good absolute fit to the data CFI = .992, TLI = .991, RMSEA = .045, SRMR = .031. Consistent with our predictions, dispositional greed significantly accounted for variance in PGSI scores, holding impulsiveness and sociodemographic variables constant. We found a similar pattern of results for the behavioral indicators of gambling (see Figure 1B), which also showed good absolute fit to the data CFI = .980, TLI = .952, RMSEA = .046, SRMR =.022, in which dispositional greed and motor impulsiveness were associated with mean gambling frequency and poly-gambling activities, but neither trait significantly predicted the amount spent within the past 14 days.

**Figure 2.**
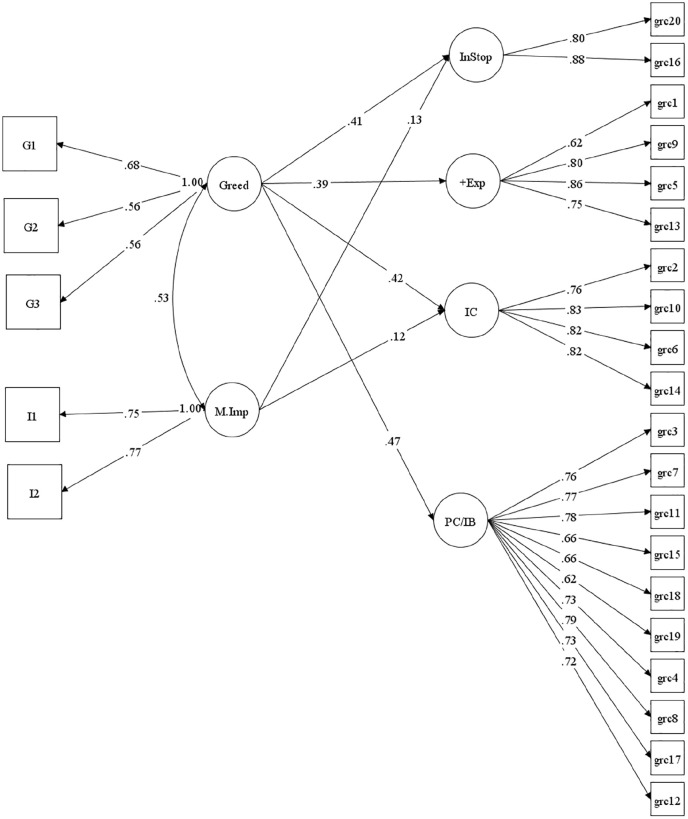
SEM Analyses for Gambling-Related Cognitions in Study 2. *Note*. InStop = Inability to Stop; +Exp = Gambling Expectancies; IC = Illusion of Control; PC/IB = Predictive Control/Interpretive Bias.

Subsequently, we focused on the degree to which dispositional greed uniquely accounted for variance in GRCs. Figure 2 shows the significant path coefficients for this model (see Supplemental Online information Table SI-3 for parameter estimates).^6^ Model fit statistics showed a good absolute fit to the data CFI = .958, TLI = .952, RMSEA = .041, SRMR = .030.

For all four GRC factors, greed showed stronger relationships between the GRCs than did motor impulsiveness. Individuals reported higher greed scores were more likely to report more maladaptive cognitive distortions, attach more positive expectancies in gambling, and report a greater perceived inability to stop gambling. These effects were present beyond that explained by trait motor impulsiveness.

#### Exploratory Analyses

We aimed to further characterize how those higher in dispositional greed, and who also gambled, thought about gambling and other decision-making constructs (see Table 3). Greedier individuals were not only more confident that they will win more than others, but also more likely to tell others about wins when they occur. They were also more likely to chase losses, and they report greater “resilience” with losses (e.g., shrugging off losses, laughing about them, etc.). Consistent with past research, we found that greedier individuals were more likely to self-report that they were a risk-taker. When examining the associations between greed and financial motivation questions, we found that gambling because of a “fear of missing out” on a win was more strongly associated with greed than a motivation solely to earn money. Finally, dispositional greed was associated with greater dispositional regret with respect to the decisions that they have made.

**Table 3.** Exploratory Analyses for Study 2.

Item	Greed *r*	Partial *r* (impulsiveness)
***Winning attitudes***		
I expect to win more than lose when gambling over the long-run	.29**	.26**
I make sure to tell others when I have won gambling	.27**	.22**
My past wins prove that I will be successful at gambling in the long-run	.31**	.27**
***Chasing losses and loss attitudes***		
If I have already lost that day, making more risky bets sounds like the best way to break-even or get ahead	.36**	.29**
If I lose one gamble, it is best to double the wager the next time	.37**	.31**
If I would lose a gamble, I would bet more next time to break-even	.34**	.28**
(Positive) Overall attitudes toward losses	.25**	.21**
***Financial motivations***		
I gamble to win money	.06**	.05**
I gamble because worried about not winning if I do not play	.27**	.23**
***Dispositional factors***		
Risk-taking (“In general, I would consider myself a risk-taker”)	.36**	.27**
Regret	.31**	.25**

*Note*. **p < .01. *N* = 3,193 to 3,195. Partial correlation value represents the correlation between greed and exploratory variables, controlling for both motor and attention/non-planning.

## General Discussion

Gambling is popular worldwide and continues to grow. The current study provides evidence that dispositional greed may be a contributing predictor of gambling participation, frequency, and realizing negative consequences from gambling, which have the potential to develop into a pathological disorder. These associations could not be explained by other dispositional factors within its nomological network, specifically materialism (in Study 1) and impulsiveness (in Study 2). In addition, our results provide unique insights into the beliefs that these individuals may hold, which may perpetuate, and perhaps accelerate, gambling behavior. Our results revealed that dispositional greed was associated with specific GRCs, namely, perceived inability to stop, gambling expectancies, and biased thinking. Furthermore, those reporting higher greed were more likely to hold distorted thoughts about chasing losses, gamble as a means to make financial gains (cf., Tabri et al., 2022), not miss out on potential wins and may use wins as a way to bolster status. In contrast to prior studies that examined greed and risk-taking with controlled laboratory tasks, this study demonstrates one way that greed and risk-taking may manifest in everyday life, along with the experiences of consequences associated with them.

The influential Gambling Pathways Model (Blaszczynski & Nower, 2002) suggests that the development of problem gambling, in part, results from distorted gambling-related schemas, such as illusions of control and interpretive biases. Reinforcement of these schemas lead to expectancies and continued gambling behavior, which in turn, results in a further escalation of gambling. Subsequently, loss chasing and negative consequences then become more likely to occur, giving rise to pathological gambling pathology. This model specifies three sub-types: (a) gamblers absent of psychopathology, who gamble for recreational and social purposes, but hold distorted cognitions about gambling, such as illusions of control, superstitious beliefs, and interpretive biases; (b) an “anti-social impulsive” gambler sub-type, who possess higher levels of disinhibition, dark triad traits, risk-taking, and comorbid substance use disorders, and uses gambling for meaning and purpose, as well as coping; and (c) an “emotionally vulnerable” group who gamble pathologically to avoid negative mood states, and who often have comorbid mood and anxiety disorders and gamble as a means for coping (Nower et al., 2022).

The distorted thought processes associated with illusions of control and interpretative biases provide the basis for a potential pathway for dispositionally greedy individuals to engage in problem-gambling behavior. Individuals reporting greater dispositional greed may demonstrate a more egocentric point of view, Subsequently, they may be more likely to interpret wins as internally caused, reinforcing their own perceived abilities, and also discount negative outcomes as being the result of chance events. Supporting this assertion, greedy individuals were more likely to shrug off losses when they occurred, even being more likely to have a laugh about them as being a part of gambling, rather than exhibit concern.

Although this study did not include a clinical sample, as often is the case with pathways model research, gamblers who reported higher levels of dispositional greed appear to share characteristics with the impulsive anti-social pathological gambling sub-type. However, our findings suggest that greed represents a unique factor, separate from impulsiveness, which may perpetuate gambling behavior. These gamblers also reported more positive gambling expectancies that center around improving one’s current negative mood state, suggesting coping motivations for gambling, which are part of the emotionally vulnerable sub-type. People reporting greater dispositional greed have been shown to experience lower satisfaction with life (e.g., Hoyer et al., 2025). Gambling may offer a potential way to quickly change this status, even if it may only temporarily alleviate dissatisfaction with life, and potentially may lead to subsequent compounding of difficulties. In this study, we found that greed was positively associated with gambling cognitions associated with the expectation of increasing happiness, and relieving stress.

As the behavior of gambling to cope with daily stresses becomes more frequent, losses are bound to mount. Perpetuating this cycle, reinforced by biased thinking, may eventually lead to feeling unable to stop once signs of problematic gambling have manifested. Greedier individuals may be more likely to develop these cognitions over time, perhaps after adopting other maladaptive cognitions, such as interpretive biases, illusions of control, and general expectancies about gambling. We speculate that this pattern represents a potentially harming feedback loop for these individuals. While the temporal dynamics of gambling pathology extend beyond our study, we feel that this is a fascinating avenue for future research.

However, we hesitate to classify greed as a unique factor to any one pathway. Instead, dispositional greed may be a common personality factor for the potential development of gambling problems, beginning with an increased tendency to endorse maladaptive cognitions and experiencing the excitement associated with gambling. Because greedy individuals are prone to be dissatisfied with their current status quo, and always wanting more, we speculate that the excitement of gambling may be particularly alluring. Notably, Li et al. (2019) reported structural and functional differences in greedy individuals which may indicate differences in the neural prefrontal reward and affect system associated with risk-taking (Damasio, 2006), which may contribute to their experience of unquenchable desire and dissatisfaction.

Although this study provides converging evidence across samples, measures, and countries, that dispositional greed may be associated with gambling behavior, and subsequent negative consequences, we do acknowledge some potential limitations and avenues for future research. First, the absence of a clinical sample of pathological gamblers leaves questions regarding the associations between greed and the experience of gambling-related harms in a clinical population unanswered. That is, although our results align with clinical studies, they are silent to clinical assessments of severity, not to mention other contributing background factors, such as prior childhood maltreatment and the presence of both internalizing and externalizing psychopathologies (Nower et al., 2022).

Related, Study 2 involved convenience online sampling, which may yield over-reporting of gambling problems in general populations (Pickering & Blaszczynski, 2021). To address this concern, we followed practices, such as maintaining age/gender/region quotas, identifying psychometrically problematic responses, as well as pre-registered the study design. Even if over-reporting still occurred, it could not readily explain the associations between greed and gambling that we observed. In contrast, Study 1 used a more clinically oriented assessment of problem gambling (i.e., SOGS) in a non-clinical sample. In combination with a much smaller engagement with gambling in Dutch culture (at the time of study), the use of the SOGS may have resulted in even lower observed base rates, not only compared with Study 2, but also the vast majority of other studies. However, it is important to note that our aim was not to provide prevalence estimates for a given population.

Second, although our results provide evidence that greed predicts gambling behavior and related cognition beyond that explained by trait impulsiveness, we acknowledge that our study only considered certain aspects of impulsiveness. While we found evidence for our hypothesis and that prior research has suggested that motor impulsiveness is higher in Gambling Disorder than in other externalizing disorders (Reid et al., 2014), this study was silent regarding other processes that may yield impulsive behavior, and subsequently, problematic gambling. For instance, impulsiveness-related traits related to rash, emotion-based decision-making (i.e., positive and negative urgency, as measured by the Urgency (both positive and negative), (lack of) Premeditation, (lack of) Perseverence, and Sensation Seeking, or UPPS-P scale, Lynam et al., 2006), have been shown to be associated with gambling behavior, and more broadly, psychopathology (Berg et al., 2015; Cyders et al., 2007, 2014). Positive urgency has been associated with levels of gambling, and some GRCs (Cyders et al., 2007; Cyders & Smith, 2008), though it should also be noted that urgency dimensions show moderate correlations with motor impulsiveness (Sharma et al., 2013; Vergés et al., 2019). Although positive urgency may account for variance in gambling behavior beyond that explained by the impulsiveness measures used in this study, we would not expect that greed and positive, or negative urgency would share more overlapping variance than what was observed. Nonetheless, past investigations between greed and impulsiveness have not been comprehensive, and we encourage future research to examine further this open question.

These considerations aside, the current research has provided insight into how dispositional greed may be an important factor in predicting adverse consequences associated with gambling. In sum, our findings were robust across measures and cultures with varying degrees of gambling participation. We hope that future endeavors will help to elucidate psychological mechanisms that greedy individuals may employ as they perpetuate their acquisition-dissatisfaction cycle. Such insights may lead to interventions for those who have experienced negative consequences from these tendencies, whether they arise from gambling, or spending time and effort to accumulate things that they no longer desire once they possessed.

## Supplemental Material

sj-docx-1-psp-10.1177_01461672251315200 – Supplemental material for Hungry Ghosts Eat Casino Chips: Associations Between Dispositional Greed and Gambling Supplemental material, sj-docx-1-psp-10.1177_01461672251315200 for Hungry Ghosts Eat Casino Chips: Associations Between Dispositional Greed and Gambling by Joshua Weller, Marcel Zeelenberg and Barbara Summers in Personality and Social Psychology Bulletin

## Notes

1. Study materials data, codebooks, and analysis code are available for both studies at Open Science Framework: https://osf.io/y85jr/.

2. Participants completed the Life Orientation Test-Revised (Scheier et al., 1994) as a measure of dispositional optimism, *M* = 3.04, *SD* = 0.51, Cronbach’s α = .78. However, our analyses found that it was not associated with either greed, any gambling cognitions, nor did it show any systematic associations with the gambling indicators. Thus, despite its inclusion in our preregistration, we did not include it here.

3. See supplementary online information Table SI-1 for EFA results. Because we only included five items, we acknowledge that it is possible, that with the full scale, the three-factor correlated structure found by Coutlee et al. (2014) would emerge (though a two-factor solution was not reported in that study).

4. Because this study was conducted with the general population in mind, we chose not to ask items that we deemed were better suited for disordered gambling populations.

5. Our pre-registered hypotheses were focused on a path-modeling approach that tested the degree to which the relationship between dispositional greed and gambling outcomes were statistically mediated by GRCs. However, upon suggestions during the review process highlighting potential concerns associated with mediation models in cross-sectional survey design, we have modified our approach. The path-model results can be found at https://osf.io/y85jr/.

6. We first tested a model with all five GRCs as correlated latent variables. However, the interpretive bias latent variable had a linear dependency with the predictive control latent variable. We addressed this by creating a latent variable that included items from both scales, and thus, we proceeded with a correlated, four-factor model.
