# Supplementary material for: Hungry Ghosts Eat Casino Chips: Associations Between Dispositional Greed and Gambling
Source: Pers Soc Psychol Bull. 2025 Mar 12;52(6):1596–612. doi: 10.1177/01461672251315200 (PMC13096610; doi:10.1177/01461672251315200)
Supplement: sj-docx-1-psp-10.1177_01461672251315200 – Supplemental material for Hungry Ghosts Eat Casino Chips: Associations Between Dispositional Greed and Gambling [file sj-docx-1-psp-10.1177_01461672251315200.docx]

SUPPLEMENTARY INFORMATION

**Loss Attitude Scale: 4-point scale (1= strongly disagree; 4 strongly agree)**

Gambling losses are only minor setbacks.

Regardless of my losses, I will eventually come out ahead.

I tend to forget about my previous losses quickly.

I joke with others about times when I’ve lost gambling.

Losses are not very stressful or upsetting to me.

In the end, losses make you a better gambler.

Table SI-1. EFA results for Impulsiveness

|  | | |
| --- | --- | --- |
|  | Factor | |
|  | 1 | 2 |
| .is self-controlled | .758 |  |
| ...concentrates easily. | .646 |  |
| …plans trip well ahead of time | .419 |  |
| ...acts on impulse |  | -.768 |
| ...says things without thinking |  | -.739 |
| Extraction Method: Principal Axis Factoring. Rotation Method: Oblimin with Kaiser Normalization. | | |

**Table SI-2.** *Parameter Estimates for SEM Models Predicting Gambling Outcomes - Study 2.*

| **Model** | | **Ustd. Estimate (S.E.)** | **Std. Estimate (S.E.)** | **95% CI for std. effect (low, high)** | |
| --- | --- | --- | --- | --- | --- |
| **PGSI Total Score**  **PGSI** | |  |  |  |  |
| PGSI 1 | | .70^**^ (.01) | .90^**^ (.01) | .88, .91 |  |
| PGSI 2 | | .69^**^ (.01) | .89^**^ (.01) | .88, .91 |  |
| PGSI 3 | | .69^**^ (.01) | .89^**^ (.01) | .88, .90 |  |
| PGSI 4 | | .72^**^ (.01) | .92^**^ (.01) | .91, .93 |  |
| PGSI 5 | | .69^**^ (.01) | .89^**^ (.01) | .88, .90 |  |
| PGSI 6 | | .72^**^ (.01) | .93^**^ (.01) | .92, .94 |  |
| PGSI 7 | | .73^**^ (.01) | .94^**^ (.01) | .93, .95 |  |
| PGSI 8 | | .73^**^ (.01) | .94^**^ (.01) | .93, .94 |  |
| PGSI 9 | | .73^**^ (.01) | .94^**^ (.01) | .93, .95 |  |
| **Greed** | |  |  |  | |
| Item 1 | | .53^**^ (.03) | .55^**^ (.02) | .51, .59 | |
| Item 2 | | .81^**^ (.03) | .72^**^ (.02) | .67, .76 |  |
| Item 3 | | .51^**^ (.03) | .45^**^ (.02) | .41, .49 |  |
| **Impulsiveness-Motor** | |  |  |  |  |
| Item 1 | | .87^**^ (.03) | .77^**^ (.02) | .74, .81 |  |
| Item 2 | | .80^**^ (.03) | .75^**^ (.02) | .71, .78 |  |
| **Impulsiveness- A/NP** | |  |  |  |  |
| Item 1 | | .55^**^ (.03) | .56^**^ (.02) | .52, .61 |  |
| Item 2 | | .74^**^ (.03) | .82^**^ (.03) | .77, .88 |  |
| Item 3 | | .41^**^ (.02) | .43^**^ (.02) | .39, .47 |  |
| **…🡪 PGSI:** | |  |  |  |  |
| Greed | | .36^**^ (.05) | .28^**^ (.04) | .20, .35 |  |
| Imp-Motor | | .30^**^ (.04) | .23^**^ (.03) | .17, .29 |  |
| Imp- A/NP | | -.03 (.03) | -.03 (.03) | -.08, .03 |  |
| Age | | -.03^**^ (.00) | -.32^**^ (.03) | -.37, -.26 |  |
| Gender | | -.43^**^ (.06) | -.17^**^ (.02) | -.21, -.12 |  |
| HH Income level | | -.07^**^ (.03) | -.06^**^ (.02) | -.10, -.02 |  |
| **Gambling Indicators (F(q)/Poly-gambling/ £spent)**: | | | |  | |
| **Greed** |  | |  |  |  |
| Item 1 | .66^**^  (.02) | | .69^**^  (.02) | .66, .73 |  |
| Item 2 | .61^**^  (.02) | | .54^**^  (.02) | .50, .58 |  |
| Item 3 | .65^**^  (.02) | | .58^**^  (.02) | .54, .61 |  |
| **Impulsiveness-Motor** |  | |  |  |  |
| Item 1 | .85^**^  (.02) | | .75^**^  (.02) | .71, .79 |  |
| Item 2 | .82^**^  (.02) | | .77^**^  (.02) | .73, .81 |  |
| **…🡪 Mean Gambling Frequency:** | | |  |  |  |
| Greed | | .19^**^  (.03) | .20^**^  (.03) | .14, .26 |  |
| Imp.-Motor | | .14^**^  (.03) | .14^**^  (.03) | .09, .19 |  |
| Age | | -.01^**^ (.03) | -.19^**^ (.02) | -.23, -.15 |  |
| Gender | | -.30^**^ (.03) | -.15^**^ (.02) | -.18,-.12 | |
| HH Income Level | | .05^**^ (.02) | .06 (.02) | .02, .09 | |
| **…🡪 Polygambling:** | |  |  |  | |
| Greed | | .36^**^ (.08) | .13^**^ (.03) | .07, .19 |  |
| Imp.-Motor | | .40^**^ (.08) | .15^**^ (.03) | .10, .20 |  |
| Age | | -.05^**^  (.00) | -.30^**^  (.02) | -.34, -.26 |  |
| Gender | | -.49^**^  (.09) | -.09^**^ (.02) | -.12,-.06 |  |
| HH Income Level | | .16^**^  (.05) | .04^**^  (.02) | .03, .10 |  |
| **…🡪 £ spent/ 14 days:** | |  |  |  |  |
| Greed | | 7.12 (6.98) | .05 (.05) | -.04, .13 |  |
| Imp.-Motor | | 3.75 (5.73) | .02 (.04) | -.05, .10 |  |
| Age | | -1.23^**^ (.30) | -.13^**^ (.03) | -.18, -.08 |  |
| Gender | | -33.08^**^  (3.32) | -.11^**^(.02) | -.15, -.06 |  |
| HH Income Level | | 5.78 (3.32) | .04 (.02) | -.01, .09 |  |

Note. ***p*<.01. *N*=3207.

**Table SI-3.** *Parameter Estimates for SEM Models Predicting Gambling Related Cognitions*

| **Model** | | **Ustd. Estimate (S.E.)** | **Std. Estimate (S.E.)** | **95% CI for std. effect (low, high)** | |
| --- | --- | --- | --- | --- | --- |
| **GRCs**  I**nability to Stop** | |  |  |  |  |
| Item 20 | | .61^**^ (.01) | .80^**^ (.01) | .78, .83 |  |
| Item 16 | | .70^**^ (.01) | .88^**^ (.01) | .87, .90 |  |
| **Expectancies** | |  |  |  |  |
| Item 1 | | .47^**^ (.01) | .62^**^ (.01) | .60, .65 |  |
| Item 9 | | .65^**^ (.01) | .80^**^ (.01) | .78, .82 |  |
| Item 5 | | .70^**^ (.01) | .86^**^ (.01) | .84, .87 |  |
| Item 13 | | .63^**^ (.01) | .75^**^ (.01) | .73, .77 |  |
| **Illusion of Control** | |  |  |  |  |
| Item 2 | | .59^**^ (.01) | .76^**^ (.01) | .74, .78 |  |
| Item 10 | | .62^**^ (.01) | .83^**^ (.01) | .82, .85 |  |
| Item 6 | | .63^**^ (.01) | .82^**^ (.01) | .80, .83 |  |
| Item 14 | | .64^**^ (.01) | .82^**^ (.01) | .80, .84 |  |
| **Predictive Control/Int. Bias** | | |  |  |  |
| Item 3 | | .55^**^ (.01) | .76^**^ (.01) | .74, .78 |  |
| Item 7 | | .60^**^ (.01) | .77^**^ (.01) | .75, .79 |  |
| Item 11 | | .59^**^ (.01) | .78^**^ (.01) | .76, .79 |  |
| Item 15 | | .54^**^ (.01) | .66^**^ (.01) | .63, .68 |  |
| Item 18 | | .55^**^ (.01) | .66^**^ (.01) | .63, .68 |  |
| Item 19 | | .48^**^ (.01) | .62^**^ (.01) | .59, .64 |  |
| Item 4 | | .57^**^ (.01) | .73^**^ (.01) | .71, .75 |  |
| Item 8 | | .59^**^ (.01) | .79^**^ (.01) | .77, .80 |  |
| Item 17 | | .60^**^ (.01) | .73^**^ (.01) | .71, .75 |  |
| Item 12 | | .57^**^ (.01) | .72^**^ (.01) | .70, .74 |  |
| **Greed** | |  |  |  | |
| Item 1 | | .65^**^ (.02) | 68^**^ (.02) | .64, .72 | |
| Item 2 | | .64^**^ (.03) | .56^**^ (.02) | .52, .61 |  |
| Item 3 | | .63^**^ (.03) | .56^**^ (.02) | .52, .60 |  |
| **Impulsiveness-Motor** | |  |  |  |  |
| Item 1 | | .84^**^ (.02) | .75^**^ (.02) | .71, .83 |  |
| Item 2 | | .83^**^ (.02) | .77^**^ (.02) | .87, .90 |  |
| **…🡪Inability to Stop:** | |  |  |  |  |
| Greed | | .48^**^ (.05) | .41^**^ (.04) | .35, .48 |  |
| Imp-Motor | | .15^**^ (.04) | .13^**^ (.03) | .07, .19 |  |
| **…🡪Expectancies** | |  |  |  |  |
| Greed | | .43^**^ (.05) | .39^**^ (.03) | .32, .46 |  |
| Imp-Motor | | .08 (.04) | .08 (.03) | .01, .14 |  |
| **…🡪Illusion of Control:** | |  |  |  |  |
| Greed | .49^**^  (.04) | | .42^**^  (.03) | .36, .49 |  |
| Imp-Motor | .14^**^  (.04) | | .12^**^  (.03) | .06, .18 |  |
| **…🡪Pred.Cont/Int.Bias** |  | |  |  |  |
| Greed | .55^**^  (.04) | | .47^**^  (.03) | .41, .53 |  |
| Imp-Motor | .10^**^  (.04) | | .08^**^  (.03) | .02, .14 |  |
|  | |  |  |  |  |
